# Trajectories of device-assessed physical activity and sleep in ICU- and non-ICU-treated patients up to 2 years after hospitalization for COVID-19, and their association with health-related quality of life

**DOI:** 10.1007/s11136-025-04095-7

**Published:** 2025-11-28

**Authors:** J. C. Berentschot, G. W. M. Broeren, L. M. Bek, M. E. Hellemons, J. van Bommel, J. G. J. V. Aerts, G. M. Ribbers, J. B. J. Bussmann, M. H. Heijenbrok-Kal, H. J. G. van den Berg-Emons

**Affiliations:** 1 Department of Respiratory Medicine, Erasmus MC, University Medical Center, Rotterdam, The Netherlands; 2 Department of Rehabilitation Medicine, Erasmus MC, University Medical Center, Rotterdam, The Netherlands; 3 Department of Intensive Care, Erasmus Medical Center, Rotterdam, The Netherlands; 4 Rijndam Rehabilitation, Rotterdam, The Netherlands; 5 Department of Respiratory Medicine, Franciscus Gasthuis & Vlietland, Rotterdam, The Netherlands; 6 Department of Respiratory Medicine, Albert Schweitzer Hospital, Dordrecht, The Netherlands; 7 Department of Adult Intensive Care Medicine, Erasmus MC, University Medical Center, Rotterdam, The Netherlands; 8 Department of Respiratory Medicine, IJsselland Hospital, Capelle aan den IJssel, The Netherlands; 9 Department of Respiratory Medicine, Ikazia Hospital, Rotterdam, The Netherlands; 10 Departments of Pediatrics and Pediatric Surgery, Intensive Care Unit, Erasmus MC Sophia Children’s Hospital, Rotterdam, The Netherlands; 11 Department of Internal Medicine, Section Nursing Science, Erasmus MC, Erasmus University Medical Center, Rotterdam, The Netherlands; 12 Department of Respiratory Medicine, Reinier de Graaf Gasthuis, Delft, The Netherlands; 13 Aafje Nursing Home, Rotterdam, The Netherlands; 14 Department of Physical Therapy, Erasmus MC, University Medical Center, Rotterdam, The Netherlands; 15 Laurens Intermezzo, Rotterdam, The Netherlands

**Keywords:** COVID-19, Accelerometer, Physical activity, Sleep, Intensive care unit, HRQoL

## Abstract

**Purpose:**

Severe COVID-19 may have lasting effects on physical and sleep behaviors, which could affect health-related quality of life (HRQoL). This study aimed to assess 2-year trajectories of device-assessed physical and sleep behaviors post-hospitalization, comparing ICU- and non-ICU-treated patients, and to assess their association with HRQoL.

**Methods:**

7-day wrist-worn accelerometer assessment at 3–6, 12, and 24 months post-hospitalization. Physical behavior: physical activity volume, time spent in light (LIPA) and moderate-to-vigorous (MVPA) physical activity, and inactivity; sleep behavior: sleep duration, efficiency, and sleep regularity index (SRI); HRQoL: EQ-5D-5L questionnaire. Multivariable generalized estimating equations models were used to assess trajectories, and associations of physical activity (LIPA, MVPA) and sleep duration with HRQoL adjusted for covariables.

**Results:**

358 patients (mean age 59.7 ± 10.5 years, 246 [69%] males, 137 [38%] ICU-treated) were included. In the total cohort, estimated mean physical activity volume was 23.5 mg/day (SE 0.40), with 153.2 min (SE 2.6) LIPA, 32.7 min (SE 1.4) MVPA, and 10.8 h (SE 0.07) inactive at 3–6 months. The estimated mean sleep duration was 6.9 (SE 0.05) hours/night, efficiency 71.1% (SE 0.41), and SRI 52.7% (SE 0.72). ICU-treated patients showed significantly lower physical activity volume (22.6 [SE 0.55] vs. 24.2 [SE 0.54], *p* = 0.05) and SRI (50.3 [SE 1.2] vs. 53.2 [SE 0.95], *p* = 0.04) compared to non-ICU-treated patients. Over time, physical and sleep outcomes remained stable in the total cohort. However, ICU-treated patients showed greater improvements in physical activity between 3–6 months and 1 year compared to non-ICU-treated patients (e.g., mean difference in change in MVPA = 6.9 min [95% CI 2.0 to 11.8], *p* = 0.006). Trajectories did not differ significantly between groups thereafter. MVPA, but not LIPA and sleep duration, was significantly associated with HRQoL, independent of covariables (β_adjusted_ 0.06 [95% CI 0.02 to 0.10], *p* = 0.004).

**Conclusion:**

Device-assessed physical and sleep behaviors seemed generally sufficient and remained stable up to 2 years post-hospitalization. However, ICU-treated patients initially showed less physical activity, reaching the levels of non-ICU-treated from the 1-year visit onwards, likely reflecting recovery from critical illness. Less time spent in MVPA was associated with poorer HRQoL; however, physical activity support should be personalized and cautiously considered in COVID-19 aftercare.

**Trial registration:**

This study has been prospectively registered in the International Clinical Trial Registry Platform (NL8710).

**Supplementary Information:**

The online version contains supplementary material available at 10.1007/s11136-025-04095-7.

## Introduction

Many patients hospitalized for COVID-19 experience long-lasting health problems, persisting for months or even years after the acute infection [1–3]. The symptoms are diverse and often co-occurring across the physical, cognitive, and psychological domains, affecting health-related quality of life (HRQoL) [4, 5]. Although long-term health problems can occur regardless of the severity of acute COVID-19, hospitalization and intensive care unit (ICU) admission are associated with a higher risk of developing persistent symptoms [6].

Physical activity and sleep are essential components of health and well-being [7–11]. Impairments in these domains are commonly observed following prolonged hospitalization and ICU treatment [12]. Indeed, hospitalization for COVID-19 is associated with an increased risk of developing long-term health problems [6]. It may be hypothesized that the burden of persistent symptoms after hospitalization for COVID-19 also affects physical and sleep behaviors [13], further deteriorating health and HRQoL. Several studies have reported reduced physical activity levels and poor sleep quality in this population [14, 15]. Gaining deeper insights into physical activity and sleep after hospitalization may yield valuable information, as these behaviors are potentially treatable targets in COVID-19 aftercare.

Most of the studies on physical activity and sleep following COVID-19 relied on questionnaires. Despite their widely use, self-reports are prone to recall bias [16], and tend to overestimate physical activity and sleep duration compared to device-based assessments [17, 18]. Device-based instruments such as accelerometers provide objective and continuous data, offering a more valid assessment of these behaviors [17]. Notably, research has shown only weak to moderate associations between self-reported and device-assessed measures of physical activity and sleep [17–20], suggesting that these measures capture related constructs but are not interchangeable. This highlights the importance of also incorporating device-based assessments when evaluating physical activity and sleep during COVID-19 recovery.

Only a few studies have conducted device-based assessments of physical activity and sleep in patients previously hospitalized for COVID-19, which were primarily cross-sectional and within the first year after hospital discharge [13, 21–25]. Two studies reported low levels of physical activity and disrupted sleep patterns 2–7 months post-hospitalization [13, 21]. One longitudinal study reported persistent circadian alterations in ICU-treated patients up to 12 months follow-up, with only a few patients having repeated assessments [23–25]. Thus, studies employing device-based assessments of physical activity and sleep—particularly those with longitudinal follow-up—in broad cohorts of patients hospitalized for COVID-19, including ICU versus non-ICU comparisons, remain limited.

Many patients experience reduced HRQoL following hospitalization for COVID-19 [5]. HRQoL is a multidimensional concept that focusses on the perceived impact of health on an individual’s quality of life, including independent physical, emotional, and social functioning as well as general well-being [26]. As such, it is an important outcome in post-COVID-19 recovery, where persistent symptoms can affect HRQoL [4, 5]. Assessing trajectories of device-assessed physical activity and sleep after hospitalization, and their associations with HRQoL, may provide valuable insights to inform COVID-19 aftercare strategies.

Therefore, this study aimed to assess trajectories of device-assessed physical and sleep behaviors up to 2 years after hospitalization for COVID-19, including comparisons between subgroups of ICU- and non-ICU-treated patients. Additionally, we assessed whether physical activity and sleep are associated with HRQoL. We hypothesized that ICU-treated patients would exhibit poorer physical and sleep behaviors than non-ICU-treated patients after hospitalization. Second, we hypothesized that poorer physical activity and sleep are associated with worse HRQoL.

## Methods

### Study design and participants

The “COVID-19 Follow-up care paths and Long-term Outcomes Within the Dutch health care system” (CO-FLOW) study is a two-year multicenter prospective cohort study conducted in the Rotterdam–Rijnmond–Delft region of the Netherlands. The study was performed in 7 hospitals (1 academic and 6 regional hospitals) and 3 rehabilitation centers (1 medical rehabilitation center and 2 skilled nursing facilities). Between July 2020 and October 2021, we included adult patients hospitalized with confirmed COVID-19 with sufficient knowledge of Dutch or English and within 6 months after hospital discharge. Incapacitated patients (e.g., dementia) were not included. More information about the CO-FLOW study design can be found elsewhere [27]. Here, we present longitudinal device-assessed physical and sleep behaviors in a subsample of the CO-FLOW study participants.

### Procedures

Study visits were scheduled around 3, 6, 12, and 24 months after hospital discharge and, when possible, alongside the clinical follow-up for COVID-19 in the participating hospitals. Patients discharged from clinical follow-up were invited to visit the Erasmus Medical Center (MC) for the remaining study visits. We arranged a home visit for patients unwilling or unable to visit the Erasmus MC. From mid-October 2020 onward, of the 650 CO-FLOW study participants, those attending their 3- or 6-month visit were invited to wear a wrist-worn accelerometer for the next 7 consecutive days to measure physical and sleep behaviors. Invitation was based solely on the availability of devices at the time of the visit; no additional in- or exclusion criteria were applied. Patients who wore the device at the 3- or 6-month visit were repeatedly invited to wear one at the 12-month and 24-month visits. Participants received the device with instructions during the study visit or by post if they could not attend the visit. Participants were instructed to wear the device on their dominant wrist [28] immediately upon receiving it, to wear it continuously or as much as possible as non-wear will lead to invalid data. They were told they could perform all kinds of activities as the device was waterproof. The accelerometer assessment period started at 7 PM on the day of study assessments for 7 continuous days of 24 h. Participants were asked to return the device in a prepaid envelope after the 7 days**.** Baseline demographic and clinical characteristics during hospital admission were retrospectively collected from medical records at the participating facilities and during the study visits, which were stored in the Castor Electronic Data Capture system (Castor EDC, Amsterdam, the Netherlands).

### Accelerometer assessments

Participants wore a wrist-worn GENEActiv tri-axial accelerometer (GENEActiv; Activinsights Ltd, Kimbolton, Cambridgeshire, UK) on their dominant wrist for 7 consecutive days, starting at 7 PM. Data were collected at sample frequency of 100Hz and acceleration was expressed relative to gravity (g units, 1 g = 9.81 m/s^2^). Accelerometer data were extracted using GENEActiv PC software V.3.2 and processed in R using the GGIR package version 3.1-0 (https://cran.r-project.org/web/packages/GGIR/) [29–31]. Signal processing in GGIR includes auto-calibration to correct for the sensor calibration error [32] and detection of non-wear time [33]. Non-wear time on valid days was imputed using GGIR’s default setting, whereby missing data were replaced by the mean value at corresponding time points on other days for the same individual [33]. The Euclidean Norm of raw accelerations Minus One (ENMO) was used to quantify the acceleration related to the movement registered, expressed in milligravity (mg) [33]. Negative values were rounded to zero and ENMO values averaged over 5-s epochs.

Sleep episodes were identified using a validated, automated sleep detection algorithm to identify the sleep period time (SPT) window from raw accelerometer data [34]. In short, the algorithm classifies periods of sustained inactivity (no change in Z-angle [wrist rotation] > 5 degrees for ≥ 5 min) [35]. Only nights with successful identification of the SPT-window, indicated by a cleaningcode of 0 or 1, were included in our analysis.

Summary characteristics were derived from the wake-up to wake-up window. Accelerometer data were considered valid if daily wear time was ≥ 16 h (GGIR default setting) on ≥ 3 days, and if wear time during either the waking-window or the SPT-window covered at least 2/3 (i.e., < 33.3% non-wear) of the respective window on ≥ 3 days or nights [36]. Complete valid wear resulted in 6 days (between waking and sleep onset) and 7 nights (between sleep onset and waking) per participant.

#### Physical behavior

The average acceleration in mg was used as a proxy for physical activity volume. Physical behavior included physical activity and inactivity (indicative of sedentary behavior), all obtained for each waking window per day [37, 38]. Physical activity was categorized by intensity based on acceleration thresholds: light physical activity (LIPA) was defined as 40–100 mg and moderate-to-vigorous physical activity (MVPA) as ≥ 100 mg. MVPA may include activities as fast walking and running [38]. LIPA and MVPA were expressed in minutes, with MVPA accumulated in ≥ 1-min bouts, retaining activities lasting at least 1 min, for which 80% of the activity met the 100 mg threshold criteria [38, 39]. The time spent in inactivity was defined by an acceleration threshold of < 40 mg, and was expressed in hours.

#### Sleep behavior

The total sleep duration was expressed in hours, which was defined by no change in arm angle greater than 5° for ≥ 5 min. Sleep efficiency was defined as the ratio between the total sleep duration and the SPT-window duration, expressed as a percentage. The sleep regularity index (SRI) is the probability of an individual being in the same state (asleep vs. awake) at any two time points 24 h apart and was expressed in a percentage ranging between 0 (random pattern) and 100 (perfect regularity, identical days) [40].

### Patient-reported outcome measures (PROMs)

PROMs were collected during all study visits. Health-related quality of life was assessed with the five-level EuroQol five-dimensional (EQ-5D-5L) questionnaire. Patients indicated their health status across 5 domains (mobility, selfcare, usual activities, pain/discomfort, anxiety/depression), each rated on a 5-level ordinal scale (no problems, slight problems, moderate problems, severe problems, unable to/extreme problems) [41]. A summary index value was calculated using the Dutch EQ-5D-5L value set by Versteegh et al. [42] with 1 indicating perfect health, 0 indicating death, and negative values indicate health states considered worse than death. In this study, we multiplied the EQ-5D index with factor 100.

Additionally, we assessed outcomes of fatigue, cognitive failures, anxiety, and depression. Fatigue was assessed with the Fatigue Assessment Scale (FAS), a total score ranging from 0–50 [43]. Cognitive failures were assessed with the Cognitive Failures Questionnaire (CFQ), a total score ranging from 0–100 [44, 45]. Anxiety and depression were assessed with the Hospital Anxiety and Depression Scale (HADS), with the subscale scores for anxiety and depression ranging from 0–21 [46].

### Baseline characteristics

Patients’ demographic and clinical characteristics at the time of hospital admission were collected through electronic patient records and during the first study visit. These included age, sex, body mass index (BMI), migration background, education level, employment status, smoking status, comorbidities, and the in-hospital characteristics treatment for COVID-19, thrombosis, delirium, ICU treatment, and the length of stay in ICU and in the hospital. Pre-COVID-19 leisure time physical activity level (inactive, light, moderate, or vigorous) was assessed using the Saltin − Grimby Physical Activity Level Scale (SGPALS) questionnaire at the first follow-up visit to assess self-reported baseline physical activity before COVID-19 infection [47]. BMI was also measured at each study visit.

### Statistical analysis

Participants with at least one valid follow-up measurement of physical or sleep behaviors were included in the analysis. Continuous variables are presented as mean with standard deviation and median with interquartile range and categorical variables as number with percentage. Baseline characteristics—including demographics and clinical characteristics at hospital admission—were compared between ICU- and non-ICU-treated patients, as well as between participants included in the analysis and those not included (i.e., not invited for accelerometer assessments or who declined participation).

Accelerometer outcomes were averaged over the valid measured days during the 7-day assessment period to obtain a daily average for each participant. The first accelerometry assessment was conducted at either the 3- or 6-month follow-up visit, and we included the questionnaire outcomes corresponding to that visit in the analysis.

We used Generalized Estimating Equations (GEE) with repeated measurements for linear models using an unstructured working correlation matrix to assess trajectories of device-assessed outcomes of physical and sleep behaviors. GEE is a semi-parametric approach that accounts for within- and between-subject correlations and has no distributional assumptions. GEE is a well-suited model to explore patterns across subgroups of ICU- and non-ICU-treated patients, providing robust population-averaged estimated marginal means. Little’s missing completely at random test was performed for variables with missing values: employment status, education level, and the time-varying variables BMI, PROMs, and physical and sleep behaviors, showing patterns in missing data (*p* = 0.001). We addressed missing data using multiple imputation (10 datasets, fully conditional specification) under the missing-at-random assumption. Estimates were pooled using Rubin’s rules.

An overview of variables and how they were handled in the GEE models is provided in Supplementary Table S1. For the total cohort analysis, we entered time (3–6, 12, and 24 months) as a fixed factor in the GEE analysis to assess the outcomes of interest (physical and sleep outcomes) over time. To assess whether outcome trajectories differed by ICU treatment, we entered time, group (ICU or non-ICU), and their interaction (group*time) into the model, adjusting for predefined covariables: age at hospital admission, sex, migration background, and the time-varying variable BMI. Post-hoc analyses were conducted for outcomes with statistically significant trajectory differences between groups, to further explore group differences across time points. We also used linear GEE models to assess associations between physical activity (LIPA and MVPA) and sleep duration with HRQoL (EQ-5D index) over time. First, we conducted univariable models to assess the association of each time-varying variable LIPA, MVPA, and sleep duration with the EQ-5D index, including time as a covariable. These variables were then jointly included in two multivariable models to explore their independent associations with EQ-5D index, while adjusting for covariables. Model 1 included LIPA, MVPA, and sleep duration, along with a priori selected covariables: time and baseline characteristics at hospital admission, including age, sex, migration background, education level, employment status, pre-COVID-19 leisure time physical activity level, the comorbidities diabetes, cardiovascular disease, and pulmonary disease, length of hospital stay, and the time-varying covariable BMI. Model 2 extended model 1 by additionally adjusting for time-varying health outcomes: fatigue (FAS score), cognitive failures (CFQ score), anxiety (HADS-A score), and depression (HADS-D score). Model parameters are presented as estimated marginal means with standard errors (SE), estimated mean differences with 95% confidence intervals (CI) and *p*-values, or beta coefficients (β) with 95% CI and *p*-values. A *p*-value < 0.05 was considered statistically significant. All statistical analyses were performed with IBM SPSS Statistics version 28 (SPSS Inc., Chicago, IL, USA).

## Results

**Fig. 1:**
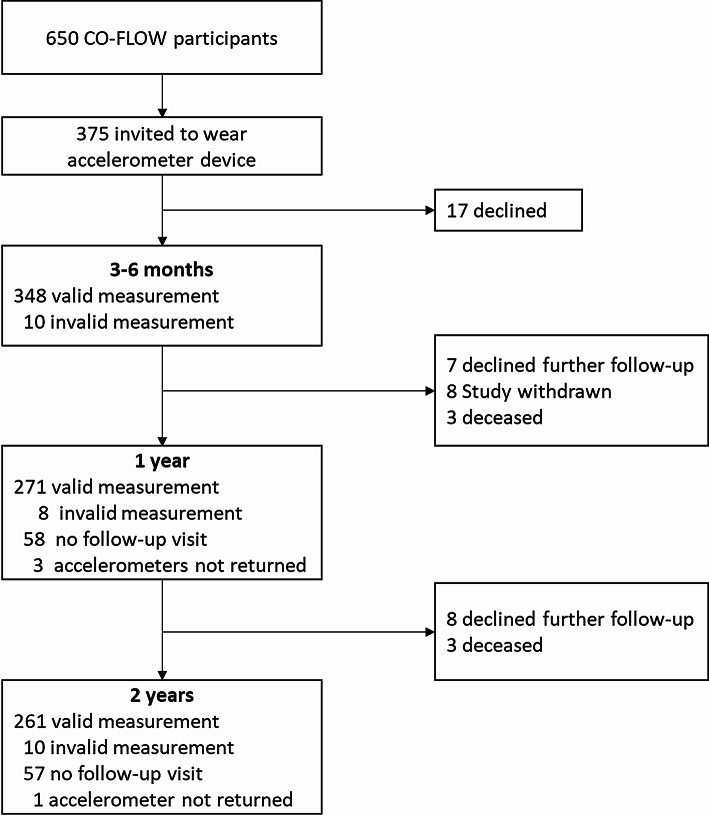
Study flowchart. In total, 358 participants had valid accelerometer data at one or more follow-up visits and were included in this study

Of the 650 CO-FLOW study participants, 375 were invited to wear an accelerometer device at the 3- or 6-month visit, of whom 17 declined. Patients with accelerometer measurements were invited for repeated measurements at the 1- and 2-year visits; however, some declined participation due to reasons such as skin rashes, inability to wear the device at work, or unwillingness to wear it. In total, 358 patients had valid accelerometer data available at one or more time points and were included in the final analysis (Fig. 1). These patients were discharged from the hospital between March 26th, 2020, and June 1st, 2021, of whom 137 (38%) patients received ICU treatment for COVID-19. Table 1 presents participants’ baseline characteristics. The mean age of all patients was 59.7 ± 10.5 years, and 246/358 (69%) were male. Regarding the 3–6-month follow-up, non-ICU-treated patients were more frequently assessed at the 3-month visit (144/221 [65%]) compared to ICU-treated patients (60/137 [44%]). Baseline characteristics were generally comparable between CO-FLOW participants with accelerometer data (n = 358) included in the analysis and those not included (n = 292, i.e., patients who were not invited or who declined the invitation), except that participants with accelerometer data were less frequently admitted to the ICU for COVID-19 (38% vs*.* 57%) (Supplemental Table S2).

**Table 1:** Baseline characteristics of study participants

	N^a^	All (n = 358)	Non-ICU (n = 221)	ICU (n = 137)
Patient characteristics				
Age, years				
Mean		59.7 ± 10.5	60.1 ± 10.2	59.2 ± 11.0
Median		60.5 (53.0–67.0)	60.0 (53.0–67.5)	61.0 (54.0–67.0)
Sex, male		246 (69%)	144 (65%)	102 (74%)
BMI, kg/m^2^	330			
Mean		29.2 (5.4)	28.7 ± 5.3	30.1 ± 5.6
Median		28.2 (25.6–32.1)	27.6 (25.2–31.3)	29.0 (26.3–33.1)
*Migration Background*				
European		266 (74%)	173 (78%)	93 (68%)
Dutch Caribbean		52 (14%)	25 (11%)	27 (20%)
Asian		17 (5%)	10 (5%)	7 (5%)
Turkish		10 (3%)	5 (2%)	5 (4%)
(North) African		13 (4%)	8 (4%)	5 (4%)
*Educational level* ^b^	356			
Low		116 (33%)	75 (34%)	41 (30%)
Middle		127 (36%)	74 (33%)	53 (39%)
High		113 (32%)	72 (33%)	41 (30%)
*Employment status*	357			
Employed		224 (63%)	137 (62%)	87 (64%)
Unemployed		54 (15%)	35 (16%)	19 (14%)
Retired		79 (22%)	49 (22%)	30 (22%)
*Smoking status*				
Never		162 (45%)	98 (44%)	64 (47%)
Former		189 (53%)	117 (53%)	72 (53%)
Current		7 (2%)	6 (3%)	1 (1%)
*Pre-COVID-19 physical activity level* ^c^				
Inactive		44 (12%)	33 (15%)	11 (8%)
Light		187 (52%)	108 (49%)	79 (58%)
Moderate		105 (29%)	68 (31%)	37 (27%)
Vigorous		22 (6%)	12 (5%)	10 (7%)
*Comorbidities*				
Obesity (BMI ≥ 30 kg/m^2^)		139 (39%)	74 (33%)	65 (47%)
Diabetes		77 (22%)	48 (22%)	29 (21%)
Cardiovascular disease or hypertension		148 (41%)	92 (42%)	56 (41%)
Pulmonary disease		83 (23%)	52 (24%)	31 (23%)
In-hospital characteristics				
*COVID-19 wave* ^d^				
First		79 (22%)	24 (11%)	55 (40%)
Second		188 (53%)	135 (62%)	53 (39%)
Third		91 (25%)	62 (28%)	29 (21%)
Thrombosis	351	63 (18%)	14 (6%)	49 (36%)
Delirium	349	85 (24%)	7 (3%)	78 (57%)
Requiring oxygen supplementation		345 (96%)	208 (94%)	137 (100%)
Requiring high flow nasal cannula		113 (32%)	39 (18%)	74 (54%)
ICU admission		137 (38%)	–	137 (100%)
Invasive mechanical ventilation		122 (34%)	–	122 (89%)
Length of ICU stay, days	135			
Mean		22.4 ± 18.6	–	22.4 ± 18.6
Median		16.0 (9.0–31.0)	–	16.0 (9.0–31.0)
Length of hospital stay, days				
Mean		19.1 ± 20.0	8.7 (6.8)	35.7 ± 22.8
Median		11.0 (6.0–27.0)	7.00 (4.0–11.0)	30.0 (19.0–48.0)
Duration between hospital discharge and accelerometer assessments				
3–6 month visit, n	348	348 (97%)	216 (98%)	132 (96%)
Days			127.7 ± 46.7	146.5 ± 46.7
Mean		134.8 ± 47.6		
Median		110.5 (92.0–184.0)	101.5 (92.0–182.0)	154.5 (98.3–189.8)
1-year visit, n	271	271 (76%)	164 (74%)	107 (78%)
Days			369.5 ± 13.2	369.5 ± 14.7
Mean		369.5 ± 13.8		
Median		366.0 (362.0–373.0)	366.0 (362.0–374.0)	366.0 (337.0–372.0)
2-year visit, days	261	261 (73%)	164 (74%)	97 (71%)
Days			741.4 ± 33.6	739.7 ± 44.3
Mean		740.8 ± 37.8		
Median		731.0 (726.0–740.0)	732.0 (726.0–741.0)	730.0 (726.0–738.0)

Data are at the time of hospital admission and presented as mean ± standard deviation, median (interquartile range), or number (%). BMI Body Mass Index, ICU Intensive Care Unit

^a^Adjusted n is presented for variables with a total number of patients less than 358

^b^Education level comprises low (primary or secondary education), middle (high school), and high (postsecondary education or university)

^c^Pre-COVID-19 leisure time physical activity level was measured with the Saltin–Grimby Physical Activity Level Scale questionnaire [37]

^d^Patients were classified by discharge date: the first COVID-19 wave (February–June 2020; original variant dominant), second wave (July 2020–January 2021; alpha variant dominant), and third wave (February–June 2021; beta and delta variants dominant)

### Device-assessed physical and sleep behaviors

#### Total cohort

**Fig. 2:**
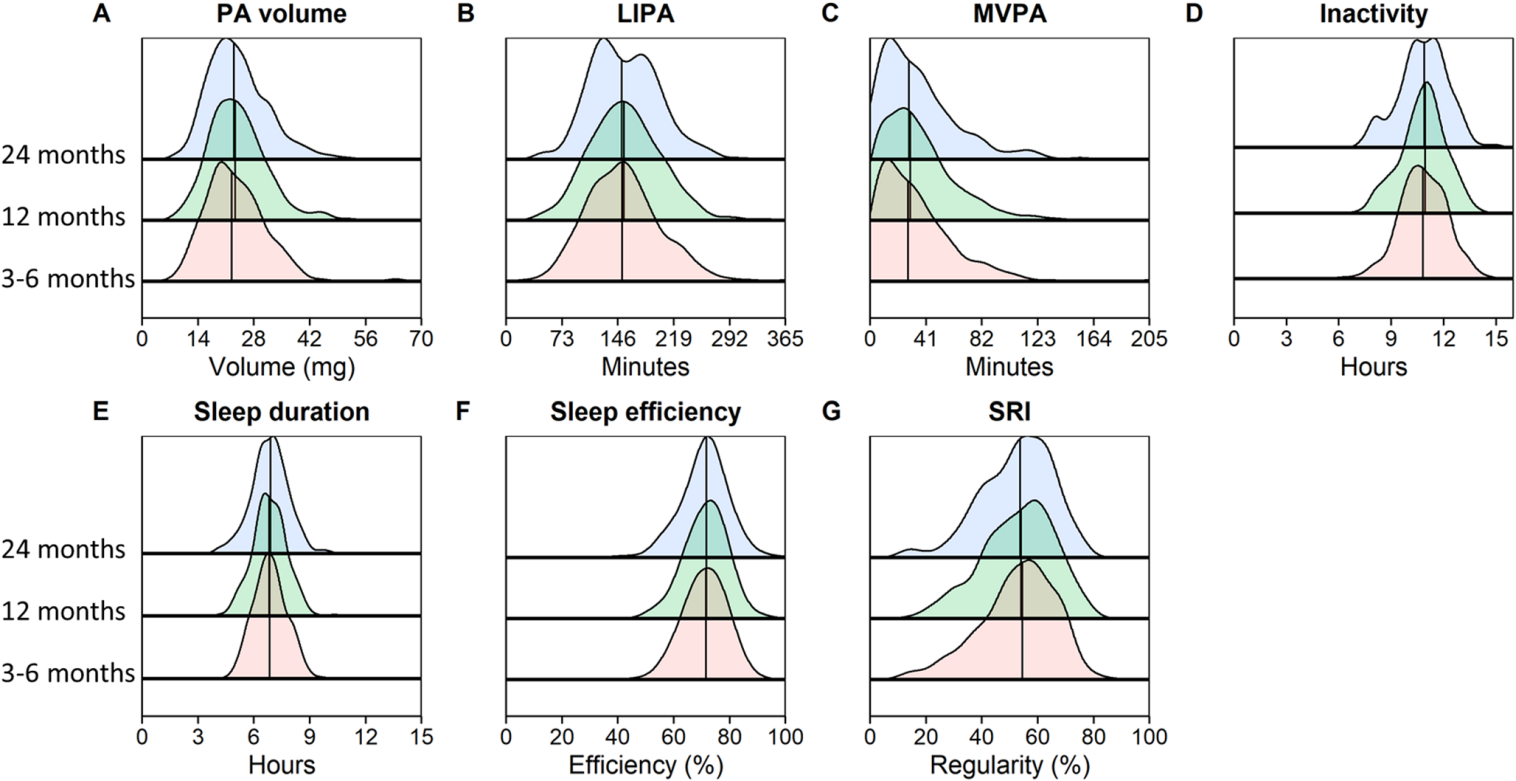
Density plots of device-assessed physical (**A**–**D**) and sleep (**E**–**G**) behaviors after hospitalization for COVID-19. Data are presented for follow-up visits at 3–6 months (red shaded area), 12 months (green shaded area), and 24 months (blue shaded area) after hospital discharge. In each density plot, the vertical lines represent the median value per visit. PA, physical activity; mg, milligravity; LIPA, light physical activity; MVPA, moderate-to-vigorous physical activity; SRI, sleep regularity index

Figure 2 shows the density plots of device-assessed physical and sleep outcomes in the total cohort at each visit (measured data). At 3–6 months, the estimated mean physical activity volume was 23.5 (SE 0.40) mg, spending 153.2 (SE 2.6) minutes in LIPA and 32.7 (SE 1.4) minutes in MVPA per day, while spending 10.8 (SE 0.07) hours inactive. The estimated mean sleep duration was 6.9 (SE 0.05) hours per night with an efficiency of 71.1% (SE 0.41) and an SRI of 52.7 (SE 0.72). Physical and sleep outcomes did not change significantly over time in the total cohort (all *p* > 0.05; Fig. 2), except for physical activity volume, which showed a significant overall time effect (*p* = 0.02); however pairwise comparisons between visits did not reach statistical significant (mean difference between 3–6 months and 1 year: 0.69 [95% CI − 0.04 to 1.4], *p* = 0.06).

#### ICU- versus non-ICU-treated patients

**Fig. 3:**
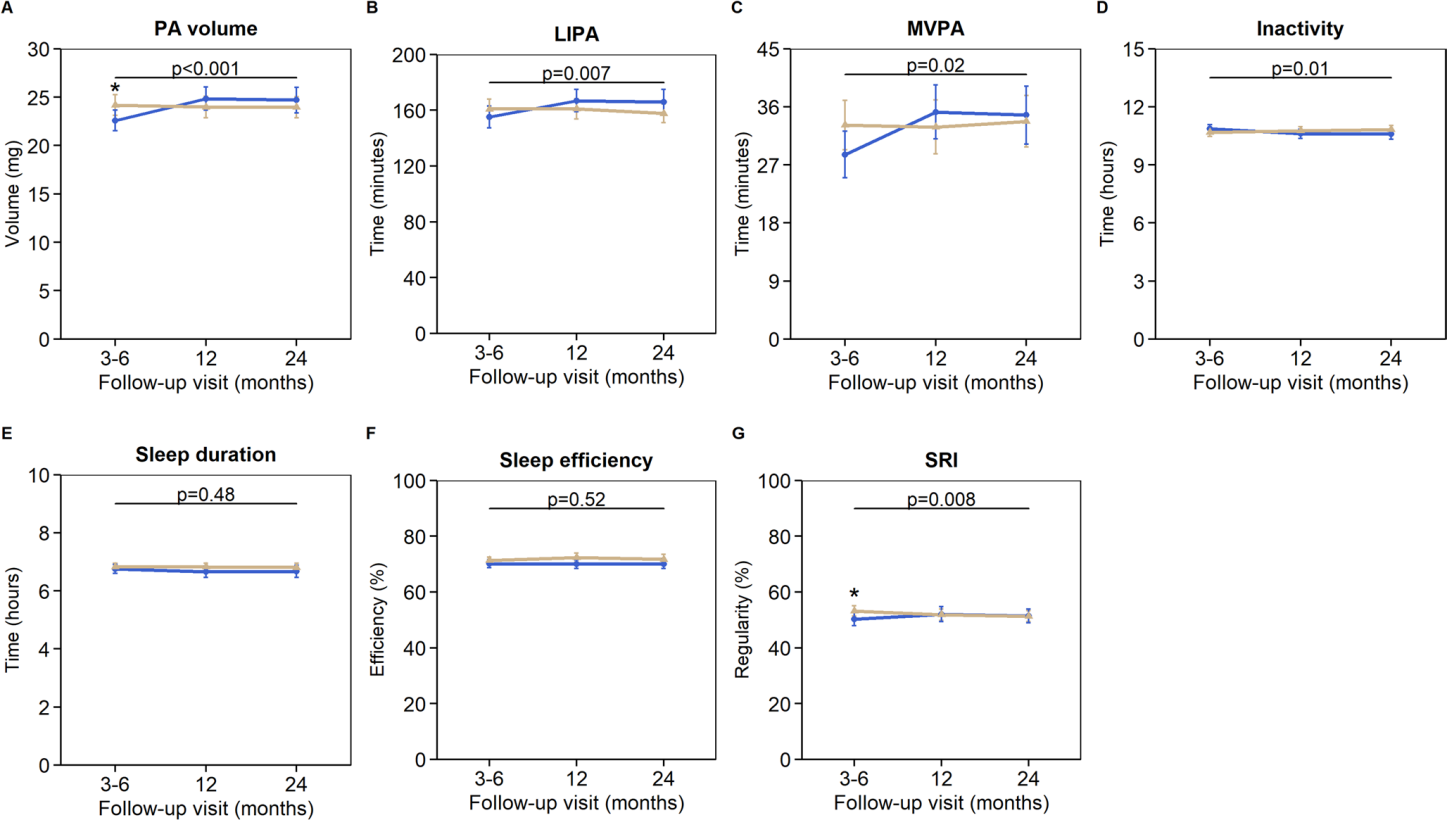
Trajectories of device-assessed physical and sleep behaviors after hospitalization for COVID-19. Data are presented as estimated mean with standard error. We used generalized estimating equations analysis to compare trajectories of physical (**A**–**D**) and sleep (**E**–**G**) variables between ICU- and non-ICU-treated patients, adjusted for age at hospital admission, sex, migration background, and time-varying BMI. *p* values for the comparison of trajectories between groups are presented in each plot; * indicates a significant (*p* < 0.05) group difference at the follow-up visit. ICU, intensive care unit; BMI, body mass index. PA: physical activity; mg, milligravity; LIPA, light physical activity; MVPA, moderate-to-vigorous physical activity; SRI, sleep regularity index

Figure 3 shows the 2-year trajectories of device-assessed physical and sleep outcomes in ICU- and non-ICU-treated patient groups. At 3–6 months follow-up, ICU-treated patients showed poorer physical outcomes, with significantly lower estimated mean physical activity volume compared to non-ICU-treated patients (22.6 [SE 0.55] vs. 24.2 [SE 0.54], *p* = 0.05). No significant group differences were observed at later follow-up visits. Between 3–6 months and 1 year, ICU-treated patients showed greater improvements in physical activity volume (mean difference 2.5 mg [95% CI 1.1 to 3.8], *p* < 0.001), time spent in LIPA (11.7 min [95% CI 0.84 to 22.5], *p* = 0.03) and MVPA (6.9 min [95% CI 2.0 to 11.8], *p* = 0.006), along with a greater reduction in inactive time (-0.34 h [95% CI − 0.62 to − 0.06], *p* = 0.02). No significant differences in trajectories were observed between the 1- and 2-year follow-up visits.

Regarding sleep outcomes, at 3–6 months follow-up, the estimated mean SRI was significantly lower in ICU-treated patients compared to non-ICU-treated patients (50.3 [SE 1.2] vs. 53.2 [SE 0.95], *p* = 0.04), while sleep duration and efficiency did not differ significantly between groups; outcomes did not differ between groups at later visits. Over time, ICU-treated patients showed a significantly greater increase in SRI compared to non-ICU-treated patients (*p* = 0.008); however, pairwise comparisons of trajectories between visits did not reach statistical significance (mean difference between 3–6 months and 1 year: 3.1% [95% CI − 0.40 to 6.6], *p* = 0.08). No significant differences were observed in the trajectories of sleep duration or efficiency.

### Association of LIPA, MVPA, and sleep duration with HRQoL

Outcomes of PROMs, including EQ-5D index, in the total cohort are shown in Supplementary Table S3; EQ-5D dimension outcomes are presented in Supplementary Figure S1. The EQ-5D index improved significantly over time in the total cohort, increasing from an estimated mean of 76.7 (SE 1.2) at 3–6 months to 79.5 (SE 1.1) at 1 year (β 2.8 [95% CI 0.34 to 5.2], *p* = 0.02); no further improvement was observed at 2 years (estimated mean 78.4 [SE 1.2]).

In univariable analysis, time spent in MVPA was significantly associated with the EQ-5D index (β_adjusted_ 0.14 [95% CI 0.09 to 0.20], *p* < 0.001), whereas LIPA (β_adjusted_ 0.03 [95% CI − 0.007 to 0.06], *p* = 0.12) and sleep duration (β_adjusted_ 0.61 [95% CI − 1.0 to 2.2], *p* = 0.46) were not. Time spent in MVPA remained independently and significantly associated with the EQ-5D index after adjusting for baseline characteristics in model 1 (β_adjusted_ 0.10 [95% CI 0.04 to 0.16], *p* = 0.001), and after further adjusting for health outcomes of fatigue, cognitive failures, anxiety, and depression in model 2 (β_adjusted_ 0.06 [95% CI 0.02 to 0.10], *p* = 0.004) (Table 2).

**Table 2:** Multivariable models for the associations between device-assessed physical activity and sleep duration with HRQoL after hospitalization for COVID-19

	Model 1 β_adjusted_ (95% CI)	*p* value	Model 2 β_adjusted_ (95% CI)	*p* value
Device-assessed variables				
LIPA, minutes	0.02 (− 0.02 to 0.06)	0.27	0.02 (− 0.008 to 0.05)	0.16
MVPA, minutes	0.10 (0.04 to 0.16)	**0.001**	0.06 (0.02 to 0.10)	**0.004**
Sleep duration, hours	0.87 (− 0.85 to 2.6)	0.32	0.71 (− 0.69 to 2.1)	0.32
Covariables				
Follow-up visit				
3–6 months	*Reference*		*Reference*	
1 year	2.5 (0.17 to 4.9)	**0.04**	1.3 (− 0.68 to 3.3)	0.19
2 years	1.4 (− 1.1 to 4.0)	0.27	− 0.40 (− 1.7 to 2.4)	0.70
Age, years	− 0.02 (− 0.23 to 0.19)	0.85	− 0.11 (− 0.25 to 0.04)	0.14
Sex				
Male	*Reference*		*Reference*	
Female	− 5.6 (− 9.5 to − 1.7)	**0.005**	− 1.9 (− 4.9 to 1.2)	0.23
BMI	− 0.20 (− 0.60 to 0.19)	0.31	− 0.08 (− 0.38 to 0.21)	0.657
Education level				
Low	*Reference*		*Reference*	
Middle	− 0.12 (− 4.1 to 3.9)	0.95	− 0.54 (− 3.5 to 2.5)	0.72
High	3.1 (− 1.0 to 7.1)	0.14	0.89 (− 2.2 to 4.0)	0.57
Employment				
Employed	*Reference*		*Reference*	
Unemployed	− 5.4 (− 10.6 to − 0.26)	**0.04**	− 6.6 (− 10.5 to − 2.6)	**0.001**
Retired	5.9 (1.5 to 10.3)	**0.008**	2.7 (− 0.42 to 5.8)	0.09
Smoking status				
Never	*Reference*		*Reference*	
Former/current	1.6 (− 1.8 to 5.1)	0.35	− 0.25 (− 2.8 to 2.3)	0.85
Migration background				
European	*Reference*		*Reference*	
Non-European	− 3.1 (− 7.0 to 0.85)	0.12	− 0.23 (− 3.3 to 2.8)	0.88
Physical activity level^a^				
Inactive/light	*Reference*		*Reference*	
Moderate/vigorous	0.61 (− 2.9 to 4.1)	0.73	− 0.66 (− 3.4 to 2.1)	0.64
Diabetes				
No	*Reference*		*Reference*	
Yes	1.4 (− 2.6 to 5.3)	0.49	2.0 (− 1.1 to 5.0)	0.21
Cardiovascular disease				
No	*Reference*		*Reference*	
Yes	− 4.3 (− 8.0 to − 0.64)	**0.02**	− 2.6 (− 5.5 to 0.29)	0.08
Pulmonary disease				
No	*Reference*		*Reference*	
Yes	− 7.9 (− 12.3 to − 3.5)	** < 0.001**	− 2.5 (− 5.6 to 0.63)	0.12
Length of stay in hospital, days	− 0.03 (− 0.11 to 0.05)	0.46	− 0.05 (− 0.11 to 0.009)	0.09
PROMs				
Fatigue (FAS total score)			− 1.1 (− 1.3 to − 0.85)	** < 0.001**
Cognitive failures (CFQ total score)			0.06 (− 0.04 to 0.16)	0.24
Anxiety (HADS-A total score)			− 0.76 (− 1.3 to − 0.31)	**0.001**
Depression (HADS-D total score)			− 0.60 (− 1.0 to − 0.16)	**0.008**

Data are presented as adjusted β-coefficient with 95% CI, obtained from generalized estimating equations analysis using linear models for HRQoL (EQ-5D-5L index multiplied with factor 100). In model 1 (n = 330), covariables included fixed patient characteristics at time of hospital admission, time-varying BMI, and the length of hospitalization for COVID-19. Model 2 (n = 324) included model 1 and further adjustment for time-varying patient-reported health outcome of fatigue, cognitive failures, anxiety, and depression. A *p* value less than 0.05 was considered statistically significant and is indicated in bold. HRQoL, health-related quality of life; EQ-5D-5L, 5-level EuroQoL-5D questionnaire; LIPA, light physical activity; MVPA, moderate-to-vigorous physical activity; BMI, body mass index; CI, confidence interval; PROMs, patient-reported outcome measures; FAS, fatigue assessment scale; CFQ, cognitive failure questionnaire; HADS, hospital anxiety and depression scale with subscale scores for anxiety (A) and depression (D)

^a^Pre-COVID-19 leisure time physical activity level was measured with the Saltin–Grimby Physical Activity Level Scale questionnairen [37]

## Discussion

This multicenter prospective cohort study assessed 2-year trajectories of device-assessed physical and sleep behaviors following hospitalization for COVID-19. Overall, these behaviors remained consistent throughout the follow-up period across the total cohort. However, ICU-treated patients had lower physical activity levels at 3–6 months follow-up, but improved over the first year post-discharge, reaching the levels of non-ICU-treated patients. While our findings preclude any causal inference, less time spent in moderate-to-vigorous physical activity (MVPA) was associated with poorer HRQoL, even after adjusting for baseline characteristics and concurrent health outcomes of fatigue, cognitive failures, anxiety, and depression.

Several studies have reported reduced physical activity levels and poor sleep quality following hospitalization for COVID-19 [14, 15]. However, these findings were predominantly based on self-reported data, which are susceptible to recall bias [16]. Devices such as accelerometers provide objective and continuous data. These measures capture related constructs but are not interchangeable [17–20]. We therefore compared our findings with previous studies that utilized device-based measures of physical activity and sleep in patients previously hospitalized for COVID-19. In one study, patients spent less time in MVPA and more time inactive at 2–7 months follow-up [13], while patients in another study were more physically active at 3–6 months follow-up [22], compared to our findings at 3–6 months after hospitalization for COVID-19. Discrepancies with previous studies may be attributed to both population-related and methodological differences. One such population-related factor is the lack of information on pre-COVID-19 physical activity levels in previous studies. Methodological variations include differences in measurement protocols, device types and placements (e.g., thigh-worn versus wrist-worn accelerometers) as well as data processing methods and classification criteria [48]. Plekhanova et al. [13] used a 14-day measurement period, whereas our study employed a 7-day protocol. Van Bakel et al. [22] categorized physical activity using metabolic equivalent of task score, while both our study and Plekhanova et al. [13] applied acceleration-based thresholds. Such methodological heterogeneity poses challenges for direct comparisons across studies.

Recently, Rowlands et al. published age-referenced values for physical activity volume using UK Biobank data [49]. Compared to its 50th percentile for the age of 60 years (28.68 mg in women; 28.09 mg in men), our patients had somewhat lower physical activity volume (approximately 24 mg/day at each visit). According to the WHO, adults are recommended to engage in at least 150–300 min of moderate-intensity physical activity or do at least 75–150 min of vigorous-intensity physical activity throughout the week for substantial health benefits, while limiting inactive time [7]. Compared to these recommendations, our data (approximately 32 min MVPA/day at each visit) suggest that patients show sufficient MVPA at the total group level.

For sleep, one study reporting device-assessed sleep behavior showed a somewhat longer sleep duration, higher sleep efficiency, and comparable SRI compared to our data at similar follow-up time after hospitalization for COVID-19 [21]. According to the American National Sleep Foundation, the recommended sleep duration is 7–9 h/night, while 6–11 h is considered ‘acceptable’; shorter or longer sleep durations are associated with negative health effects [50]. Our patients slept approximately 7 h/night, suggesting a good sleep duration.

It should be noted that most available evidence for physical and sleep guidelines comes from studies using self-reports [7, 51]. Together, it is thus challenging to draw definitive conclusions about the level of physical and sleep behaviors observed in our study. Nonetheless, our findings suggest that patients generally maintained sufficient physical activity and sleep following hospitalization for COVID-19.

ICU-treated patients for COVID-19 showed greater improvements in physical activity during the first year post-discharge than non-ICU-treated patients. This pattern aligns with our previous findings, which showed that ICU-treated patients for COVID-19 had poorer objectively assessed physical fitness at 3 months after hospitalization but improved over time, eventually reaching levels comparable to, or even exceeding, those of non-ICU-treated patients in later follow-ups [52]. These findings are clinically relevant, as ICU survivors are known to be at increased risk for new or worsening physical, cognitive, and mental health problems after discharge—a condition referred to as post-intensive care syndrome [53]. Most ICU-treated patients in our study had a good pre-COVID-19 health status, and they often were transferred to inpatient rehabilitation centers with intensive therapy [54], which may have contributed to the observed physical improvements. Our findings support the importance of the first year following ICU care as a critical period for physical recovery, as ICU-treated patients showed improvements in objectively assessed physical outcomes during this time frame. The data indicated that physical and sleep behaviors were comparable between ICU-treated and non-ICU-treated patients from the 1-year follow-up onwards, suggesting that long-term rehabilitation strategies regarding these behaviors may not need to differ between groups. Instead, rehabilitation strategies should prioritize personalized care.

Many patients experience reduced HRQoL after hospitalization for COVID-19 [4, 5, 55]. We found that less time spent in MVPA was associated with poorer HRQoL. Although the association was modest, MVPA remained independently associated with HRQoL after adjusting for baseline characteristics and concurrent health problems. Notably, over 50% of our patients experienced fatigue throughout follow-up, a symptom known to negatively impact HRQoL [4].

It is important to consider patients’ ability to engage in MVPA after COVID-19. For instance, post-exertional malaise (PEM)—a worsening of symptoms following physical, mental, or cognitive exertion—is increasingly recognized as a debilitating long-term sequelae following COVID-19, affecting HRQoL [55, 56]. PEM can be a significant barrier to physical activity, and some of our patients may have limited their MVPA during accelerometer assessments to avoid symptom exacerbation [57, 58]. Unfortunately, PEM was not assessed during our study measurements. Patients with PEM are advised to follow personalized activity programs that support engagement in physical activity without exceeding their energy limits [57, 59]. Therefore, although MVPA was positively associated with HRQoL, physical activity support should be personalized and cautiously implemented in COVID-19 aftercare.

### Strengths and limitations

Key strengths include the study’s prospective multicenter design and longitudinal 2-year follow-up period after hospitalization for COVID-19. We measured device-assessed physical and sleep behaviors, enabling objective and continuous data collection. Nonetheless, cross-study comparisons remain challenging due to methodological differences, such as measurement protocols and data processing approaches. By applying a consistent methodology, our study strengthens the validity of both longitudinal and between-group comparisons of trajectories in ICU- and non-ICU-treated patients.

The study has several limitations. It should be noted that while we observed an association between MVPA and HRQoL, our findings preclude any causal inference. We were unable to compare our outcomes with pre-COVID-19 physical and sleep behaviors. To gain some insights, we used the SGPALS to assess pre-morbid physical activity [47], despite its reliance on self-report. Our study lacks a control group of individuals without COVID-19. In addition, we lack data on the total eligible recruitment population across participating hospitals due to the surge of patients admitted to the participating centers. However, the recruitment of study participants occurred independently of the patient’s recovery status and primarily depended on the availability of research personnel. Selection bias might play a role in our study as the study is composed of a high proportion (38%) of ICU-treated patients, possibly due to the high inclusion rate from our academic hospital, compared to the average ICU admissions across all Dutch hospitals (14%). However, this allowed for comparisons between ICU- and non-ICU-treated patients. In our sample of CO-FLOW study participants with accelerometer assessments, a lower proportion had been treated in the ICU (38%) compared to those not participating in the current study (42%). This difference may be explained by the higher ICU admission rates during the first COVID-19 wave in the Netherlands (February-June 2020). CO-FLOW study measurements started in July 2020, whereas the accelerometer assessments began in mid-October 2020. We therefore missed some patients from the first wave. Additionally, ICU-treated patients were more frequently invited for accelerometer assessments at the 6-month follow-up visit rather than at 3 months, leading to a longer interval between hospital discharge and the first accelerometer assessment in this group compared to the non-ICU-treated group. Consequently, the observed differences in physical activity between ICU- and non-ICU-treated patients may be underestimated in relation to the 3-month timeframe, as earlier assessments might have captured more pronounced differences between groups.

## Conclusion

Device-assessed physical and sleep behaviors seemed generally sufficient and stable in the cohort up to 2 years after hospitalization for COVID-19. However, ICU-treated patients started with less physical activity, improving over time and reaching the levels of non-ICU-treated patients from the 1-year visit onwards, likely reflecting recovery from critical illness. Less time spent in moderate-to-vigorous physical activity was associated with poorer HRQoL, even after adjusting for covariables. However, physical activity support should be personalized and cautiously considered in COVID-19 aftercare.

## Supplementary Information

Below is the link to the electronic supplementary material.

Supplementary file1 (DOCX 265 KB)

## Data Availability

The datasets used and/or analyzed during the current study are available from the corresponding author on reasonable request.
