# Supplementary material for: Trajectories of device-assessed physical activity and sleep in ICU- and non-ICU-treated patients up to 2 years after hospitalization for COVID-19, and their association with health-related quality of life
Source: Qual Life Res. 2025 Nov 28;34(11):3191–204. doi: 10.1007/s11136-025-04095-7 (PMC12681491; doi:10.1007/s11136-025-04095-7)
Supplement: Supplementary file 1 — Supplementary file1 (DOCX 265 KB) [file 11136_2025_4095_MOESM1_ESM.docx]

**Supplementary Material**

**Trajectories of device-assessed physical activity and sleep in ICU- and non-ICU-treated patients up to 2 years after hospitalization for COVID-19, and their association with health-relate quality of life**

**Authors**

J.C. Berentschot^1^, G.W.M. Broeren^2^, L.M. Bek^2^, M.E. Hellemons^1^, J. van Bommel^3^, J.G.J.V. Aerts^1^, G.M. Ribbers^2,4^, J.B.J. Bussmann^2^, M.H. Heijenbrok-Kal^2,4^, H.J.G. van den Berg-Emons^2^, on behalf of the CO-FLOW Collaboration Group

**Affiliations**

^1^ Department of Respiratory Medicine, Erasmus MC, University Medical Center Rotterdam The Netherlands.

^2^ Department of Rehabilitation Medicine, Erasmus MC, University Medical Center Rotterdam, The Netherlands.

^3^ Department of Intensive Care, Erasmus Medical Center, Rotterdam, The Netherlands.

^4^ Rijndam Rehabilitation, Rotterdam, The Netherlands.

**Supplementary Table S1: Description of variables**

| Variables | Description |
| --- | --- |
| **Accelerometer parameters** |  |
| *Physical activity* |  |
| Average acceleration | A proxy for physical activity volume in miligraphity |
| Light-intensity physical activity | Time (in minutes) accumulated with an acceleration between 40 and 100 mg^1^ |
| Moderate-to-vigorous intensity physical activity | Time (in minutes) accumulated in bouts of 1-minute with an acceleration above 100 mg^2,3^ |
| Inactive time | Time (in hours) accumulated during the waking day below 40 mg^1^ |
| *Sleep* |  |
| Sleep duration | Time (hours) accumulated sleep within the sleep period time (SPT) window. The SPT-window is the time between the onset of sleep and the final wake-up of the night |
| Sleep efficiency | The percentage of sleep time in the duration of the SPT-window |
| Sleep regularity index | The percentage probability that a participant is in the same state (asleep or awake) at any two time points exactly 24 hours apart.^4^ A participant who is asleep/awake at the exact same times every day would score 100. |
|  |  |
| **Health-related quality of life (HRQoL)** | HRQoL was assessed with the five-level EuroQol five-dimensional (EQ-5D-5L) questionnaire.^5^ A summary index value^6^ was calculated and multiplied with factor 100 for statistical analysis. |
| **Covariables** |  |
| Age | Age (years) at time of hospital admission |
| Sex | Male or female |
| Body mass index | Body mass index (kg/m^2^) was assessed at time of hospital admission as well as during each study visit. The time-varying variable was entered in multivariable analysis |
| Migration background | Migration background was categorized into European, Dutch Caribbean, Asian, Turkish, and (North) African, collected during the first study visit. Migration background was dichotomized into European vs. non-European in multivariable analysis |
| Education level | Pre-COVID-19 education level was categorized into low (primary or secondary education), middle (high school), and high (postsecondary education or university), collected during the first study visit |
| Employment | Pre-COVID-19 employment status was categorized into employed, unemployed, and retired, collected during the first study visit |
| Smoking | Pre-COVID-19 smoking status was categorized into never, former, or current smoker, collected during the first study visit |
| Pre-COVID-19 leisure time physical activity level | Pre-COVID-19 leisure time physical activity level was measured with the Saltin–Grimby Physical Activity Level Scale questionnaire,^7^ which includes the levels: inactive, light, moderate, vigorous activity levels, collected during the first study visit. The variable was dichotomized into inactive/light vs. moderate/vigorous activity levels in multivariable analysis |
| comorbidities | Pre-COVID-19 comorbidities were collected through electronic patient records and during the first study visit. The three most common comorbidities were entered in multivariable analysis, including diabetes, cardiovascular disease, and pulmonary disease |
| length of hospital stay | Number of days in the hospital for COVID-19, collected through electronic patient records |
|  |  |
| Fatigue | Fatigue was assessed using the Fatigue Assessment Scale (FAS); the total score ranges from 0 to 50, cutoff ≥ 22.^8^ The FAS was assessed at each study visit |
| Cognitive failures | Cognitive failures were assessed using the Cognitive Failures Questionnaire (CFQ); the total score ranges from 0 to 100, cutoff >43.^9,10^ The CFQ was assessed at each study visit |
| Anxiety | Anxiety was assessed using the Hospital Anxiety and Depression Scale (HADS) questionnaire. The HADS anxiety subscale score ranges from 0 to 21, cutoff ≥ 11.^11^ The HADS was assessed at each study visit |
| Depression | Depression was assessed using the Hospital Anxiety and Depression Scale (HADS) questionnaire. The HADS depression subscale score ranges from 0 to 21, cutoff ≥ 11.^11^ The HADS was assessed at each study visit |

**Supplemental Table S2.** Baseline characteristics of CO-FLOW study participants included in the analysis and those not included, stratified for ICU treatment.

|  | Participants included in the analysis | | Participants not included in the analysis | |
| --- | --- | --- | --- | --- |
|  | Non-ICU  (n=221) | ICU  (n=137) | Non-ICU  (n=156) | ICU  (n=136) |
| **Patient characteristics** |  |  |  |  |
| Age (years)  Mean  Median | 60.1±10.2  60.0 (53.0-67.0) | 59.2±11.0  61.0 (54.0-67.5) | 61.3±12.8  61.0 (53.3-72.0) | 58.1±12.0  59.0 (53.0-66.0) |
| Sex, male | 144 (65%) | 102 (74%) | 100 (64%) | 103 (76%) |
| BMI (kg/m²), n  Mean  Median | 195  28.7±5.3  27.6 (25.2-31.3) | 135  30.1±5.6  29.0 (26.3-33.1) | 132  28.3±4.7  27.6 (25.3-30.8) | 127  30.9±5.5  30.8 (26.3-33.8) |
| *Migration Background, n* | 221 | 137 | 145 | 127 |
| European | 173 (78%) | 93 (68%) | 107 (74%) | 76 (60%) |
| Dutch Caribbean | 25 (11%) | 27 (20%) | 17 (12%) | 20 (16%) |
| Asian | 10 (5%) | 7 (5%) | 9 (6%) | 13 (10%) |
| Turkish | 5 (2%) | 5 (4%) | 8 (5%) | 10 (8%) |
| (North) African | 8 (4%) | 5 (4%) | 4 (3%) | 8 (6%) |
| *Educational level,^a^ n* | 221 | 135 | 145 | 124 |
| Low | 75 (34%) | 41 (30%) | 55 (38%) | 51 (41%) |
| Middle | 74 (33%) | 53 (39%) | 47 (32%) | 44 (36%) |
| High | 72 (33%) | 41 (30%) | 43 (30%) | 29 (23%) |
| *Employment status, n* | 221 | 136 | 156 | 125 |
| Employed | 137 (62%) | 87 (64%) | 71 (49%) | 77 (62%) |
| Unemployed | 35 (16%) | 19 (14%) | 25 (17%) | 21 (17%) |
| Retired | 49 (22%) | 30 (22%) | 49 (24%) | 27 (22%) |
| *Smoking status* | 221 | 137 | 136 | 127 |
| Never | 98 (44%) | 64 (47%) | 61 (42%) | 57 (45%) |
| Former | 117 (53%) | 72 (53%) | 82 (56%) | 68 (53%) |
| Current | 6 (3%) | 1 (1%) | 3 (2%) | 2 (2%) |
| *Pre-COVID-19 Physical activity level,*^b^ *n* | 221 | 137 | 144 | 122 |
| Inactive | 33 (15%) | 11 (8%) | 28 (19%) | 14 (12%) |
| Light | 108 (49%) | 79 (58%) | 78 (54%) | 67 (55%) |
| Moderate | 68 (31%) | 37 (27%) | 26 (18%) | 37 (30%) |
| Vigorous | 12 (5%) | 10 (7%) | 12 (8%) | 4 (3%) |
| *Comorbidities* |  |  |  |  |
| ≥1 | 179 (81%) | 112 (82%) | 124 (82%) | 119 (89%) |
| Obesity (BMI ≥30 kg/m²) | 74 (33%) | 65 (47%) | 47 (30%) | 80 (59%) |
| Diabetes | 48 (22%) | 29 (21%) | 30 (19%) | 23 (17%) |
| Cardiovascular disease  or hypertension | 92 (42%) | 56 (41%) | 54 (35%) | 55 (40%) |
| Pulmonary disease | 52 (24%) | 31 (23%) | 49 (31%) | 30 (22%) |
| Renal disease | 20 (9%) | 12 (9%) | 18 (12%) | 9 (7%) |
| Gastrointestinal disease | 7 (3%) | 7 (5%) | 13 (8%) | 4 (3%) |
| Neuromuscular disease | 27 (12%) | 10 (7%) | 15 (10%) | 16 (12%) |
| Malignancy | 28 (13%) | 13 (9%) | 12 (8%) | 16 (12%) |
| Autoimmune/inflammatory disease | 30 (14%) | 11 (8%) | 18 (12%) | 9 (7%) |
| Mental disorder | 12 (5%) | 8 (6%) | 9 (6%) | 3(2%) |
| **In-hospital characteristics** |  |  |  |  |
| *COVID-19 wave,^c^ n* | 221 | 137 | 156 | 136 |
| First | 24 (11%) | 55 (40%) | 48 (31%) | 53 (39%) |
| Second | 135 (62%) | 53 (39%) | 89 (57%) | 62 (46%) |
| Third | 62 (28%) | 29 (21%) | 19 (12%) | 21 (15%) |
| ICU admission | - | 137 (100%) | - | 136 (100%) |
| Invasive mechanical  ventilation | - | 122 (89%) | - | 113 (83%) |
| Length of ICU stay, days  Mean  Median | -  - | 22.4±18.6  16.0 (9.0-31.0) | - | 21.6±16.5  18.0 (9.0-31.0) |
| Length of hospital stay, days  Mean  Median | 8.7± 6.8  7.00 (4.0-11.0) | 35.7±22.8  30.0 (19.0-48.0) | 8.2±8.3  6.0 (4.0-10.0) | 34.8±21.2  33.0 (18.0-45.8) |
| *Baseline characteristics at the time of hospital admission are presented for participants with accelerometer data included in the analysis and those not included (i.e., patients who were not invited or who declined the invitation), stratified for ICU treatment. Data are presented as mean*±standard deviation, median (interquartile range), or *number (%). Adjusted n is presented for variables with missing values. BMI, Body Mass Index; ICU, Intensive Care Unit; NA, Not Applicable.*  *^a^ Education comprises low (primary or secondary education); middle (high school); high (postsecondary education or university).*  *^b^ Pre-COVID-19 leisure time physical activity level was measured with the Saltin–Grimby Physical Activity Level Scale questionnaire.^7^*  *^c^ Patients were classified by discharge date: the first COVID-19 wave (February–June 2020; original variant dominant), second wave (July 2020–January 2021; alpha variant dominant), and third wave (February-June 2021; beta and delta variants dominant).* | | | | |

| **Supplemental Table S3.** Health outcomes up to 2 years after hospitalization for COVID-19. | | | |
| --- | --- | --- | --- |
|  | 3-6 months | 12 months | 24 months |
| HRQoL, n | 321 | 291 | 274 |
| EQ-5D-5L index  Mean  Median | 77.3±22.1  81.9 (69.5-91.4) | 81.0±19.6  86.1 (73.0-100.0) | 80.3±20.2  84.8 (73.7-100.0) |
| Fatigue, n | 319 | 293 | 272 |
| FAS, total score  Mean  Median | 24.6±9.0  24.0 (17.0-32.0) | 23.4±8.9  22.0 (16.0-29.0) | 23.2±8.9  22.0 (16.0-30.0) |
| Fatigue  (FAS ≥ 22) | 182 (57%) | 159 (54%) | 137 (50%) |
| Cognitive failures, n | 313 | 288 | 278 |
| CFQ, total score  Mean  Median | 30.8±18.4  28.0 (16.0-44.0) | 31.2±18.1  30.0 (16.3-42.0) | 30.9±17.7  30.0 (16.8-42.0) |
| Cognitive failure (CFQ > 43) | 79 (25%) | 65 (23%) | 68 (24%) |
| Anxiety, n | 319 | 290 | 281 |
| HADS‑A, total score  Mean  Median | 5.3±4.3  5.0 (2.0-8.0) | 4.7±4.5  4.0 (1.0-7.0) | 4.5±4.2  4.0 (1.0-7.0) |
| Anxiety (HADS A ≥ 11) | 41 (13%) | 31 (11%) | 28 (10%) |
| Depression, n | 319 | 290 | 281 |
| HADS‑D, total score  Mean  Median | 4.9±4.1  4.0 (1.0-7.0) | 4.3±4.0  3.0 (1.0-7.0) | 4.4±3.9  3.0 (1.0-7.0) |
| Depression (HADS D ≥ 11) | 33 (10%) | 31 (11%) | 25 (9%) |
| *Data are presented as mean±SD, median (interquartile range) or number (%). HRQoL, Health-Related Quality of Life; EQ-5D-5L, 5-level EuroQoL-5D questionnaire; FAS, fatigue assessment scale; HADS, hospital anxiety and depression scale with subscale scores for anxiety (A) and depression (D); CFQ, cognitive failure questionnaire.* | | | |

**Supplementary Figure S1.** Proportion of responses by level of severity for EQ-5D-5L dimensions at study visits.


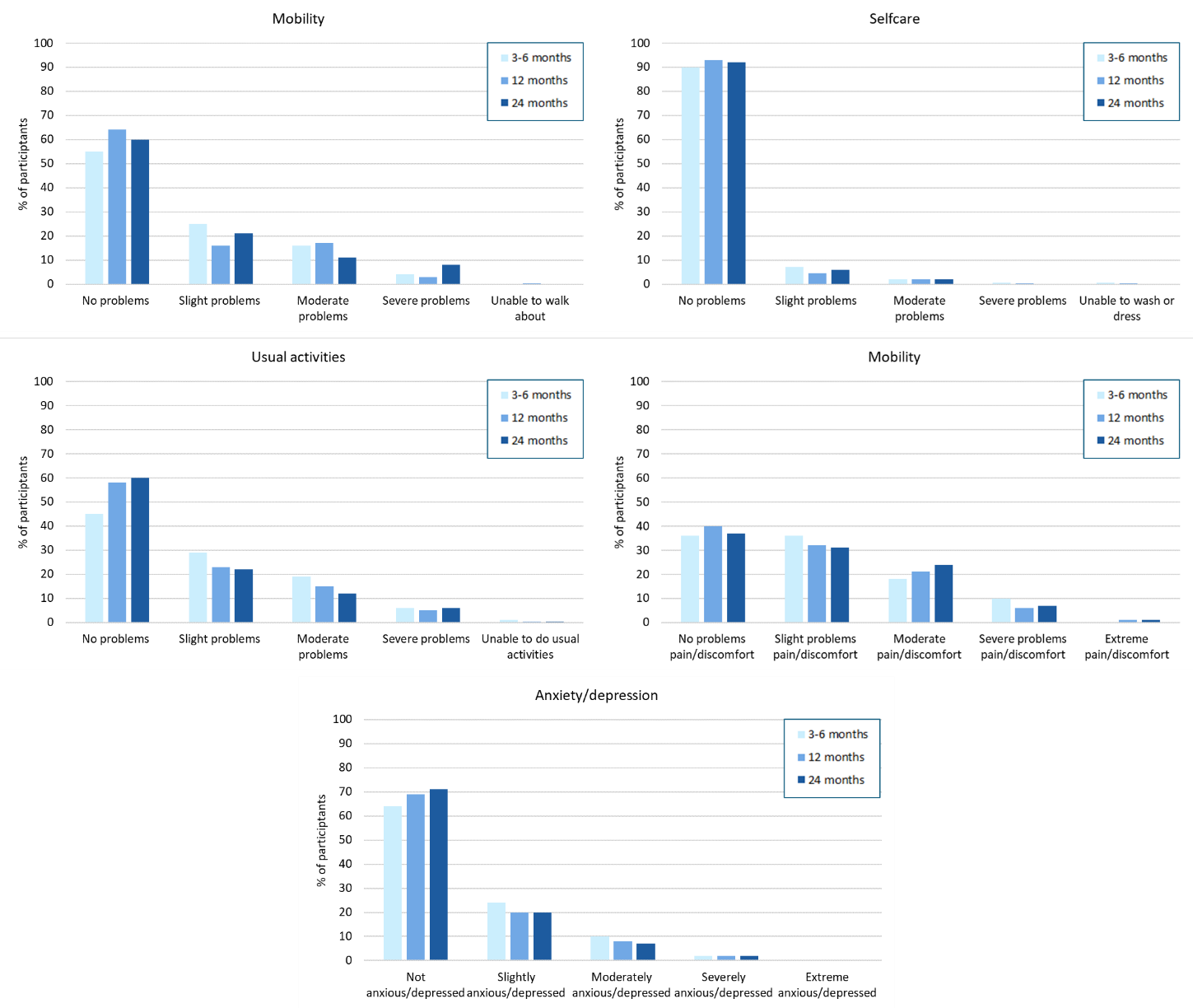


*Proportion of patients across response levels of severity for each domain of the EQ-5D-5L questionnaire. EQ-5D-5L, 5-level EuroQoL-5D questionnaire.*

**References**

1. Hildebrand M, Hansen BH, van Hees VT, Ekelund U. Evaluation of raw acceleration sedentary thresholds in children and adults. *Scand J Med Sci Sports*. 2017;27(12):1814-23.

2. Hildebrand M, VT VANH, Hansen BH, Ekelund U. Age group comparability of raw accelerometer output from wrist- and hip-worn monitors. *Med Sci Sports Exerc*. 2014;46(9):1816-24.

3. Menai M, van Hees VT, Elbaz A, et al. Accelerometer assessed moderate-to-vigorous physical activity and successful ageing: results from the Whitehall II study. *Sci Rep*. 2017;8:45772.

4. Phillips AJK, Clerx WM, O'Brien CS, et al. Irregular sleep/wake patterns are associated with poorer academic performance and delayed circadian and sleep/wake timing. *Sci Rep*. 2017;7(1):3216.

5. EuroQol G. EuroQol--a new facility for the measurement of health-related quality of life. *Health Policy*. 1990;16(3):199-208.

6. M MV, K MV, S MAAE, et al. Dutch Tariff for the Five-Level Version of EQ-5D. *Value Health*. 2016;19(4):343-52.

7. Grimby G, Börjesson M, Jonsdottir IH, et al. The "Saltin-Grimby Physical Activity Level Scale" and its application to health research. *Scand J Med Sci Sports*. 2015;25 Suppl 4:119-25.

8. de Kleijn WP, De Vries J, Wijnen PA, Drent M. Minimal (clinically) important differences for the Fatigue Assessment Scale in sarcoidosis. *Respir Med*. 2011;105(9):1388-95.

9. Broadbent DE, Cooper PF, FitzGerald P, Parkes KR. The Cognitive Failures Questionnaire (CFQ) and its correlates. *Br J Clin Psychol*. 1982;21(1):1-16.

10. Ponds R, van Boxtel, M. P. J., & Jolles, J. De Cognitive Failure Questionnaire als maat voor subjectief cognitief functioneren. *Tijdschrift voor neuropsychologie*. 2006;1(2):37-45.

11. Herrmann C. International experiences with the Hospital Anxiety and Depression Scale-a review of validation data and clinical results. *Journal of psychosomatic research*. 1997;42(1):17-41.
